# Surgical Management of Pachyonychia Congenita in a 3-Year-Old

**DOI:** 10.1055/s-0043-1771520

**Published:** 2023-12-21

**Authors:** Jack D. Sudduth, Christopher Clinker, Matthew Holdaway, Jessica L. Marquez, Jacob Veith, Thomas Wright, W. Bradford Rockwell

**Affiliations:** 1 Division of Plastic Surgery, Department of Surgery, The University of Utah Hospital, Salt Lake City, Utah

**Keywords:** pachyonychia congenita, germinal matrix excision, nail bed excision, surgical dermatology, congenital

## Abstract

Pachyonychia congenita is a rare genetic disorder characterized by hypertrophic nail plates, hyperkeratotic nail beds, and thickened hyponychium of the fingers and toes, impairing manual dexterity and resulting in poor aesthetics. The current body of literature describes various treatment modalities, but no singular approach has been defined as the gold standard. In this case, the authors employed different surgical techniques for treating pachyonychia congenita to evaluate the most effective approach. A 3-year-old boy presented with hypertrophic nail growth involving all digits of both hands and feet. Three surgical procedures were performed on the patient's fingers and toes using germinal matrix excision (GME) alone, GME plus partial sterile matrix excision (pSME), or GME plus complete sterile matrix excision (cSME). The digits treated with GME + cSME exhibited no recurrence of nail growth. Those treated with GME alone exhibited recurrence of hypertrophic nail growth, although their growth slowed. Excision of GME + cSME prevented recurrence of hypertrophic nails, while GME alone or with pSME led to slower-growing hypertrophic nails. Complete excision of the germinal and sterile matrices with skin graft closure may be a definitive treatment for pachyonychia congenita, but further studies are needed to validate these findings.

## Introduction

Pachyonychia congenita (PC) is an autosomal dominant genetic disorder characterized by hypertrophic nail plates, hyperkeratotic nail beds, and thickened hyponychium of the fingers and toes. 1 The distal two-thirds of the nail plates become thickened, elevated, and transversely arched due to hyperkeratosis of the nail bed. The thickened portion often becomes discolored, resulting in yellow or brown nails. Additional symptoms associated with PC include leukoplakia, palmar and plantar hyperkeratosis, and epidermal cysts but vary depending on the genetic variant. Management is often determined by both type and severity of the keratin genetic mutation. 1 Because of this, the importance of genetic testing prior to treatment cannot be understated.

Because of the effect PC has on the nail beds, it also impairs manual dexterity and often leads to surgical referral. Despite increased awareness and ongoing research into PC, literature regarding the surgical management of the disorder remains limited. 2 3 Many treatments for PC focus on pain management with Botox injection and sirolimus, which has shown some efficacy in managing symptoms. 1 4 We present our experience and results of treating a pediatric patient with a combination of germinal matrix excision (GME) alone, GME with partial sterile matrix excision (pSME), and GME with complete sterile matrix excision (cSME).

## Case

**Fig. 1:**
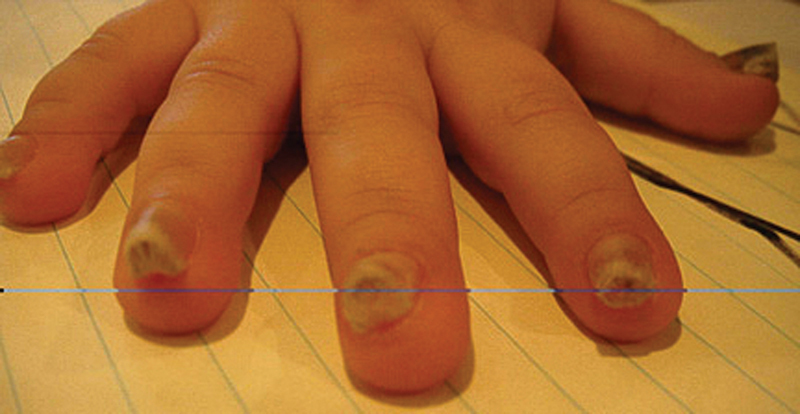
Right hand preoperatively. The nail plates show significant thickening on all five digits.

A 3-year-old boy presented with hypertrophic nail growth involving all digits of both hands and feet ( Fig. 1 ). The nails caused persistent pain and discomfort and required frequent trimming. This patient's parents opted for definitive surgical treatment. No previous genetic testing was done on the patient.

**Fig. 2:**
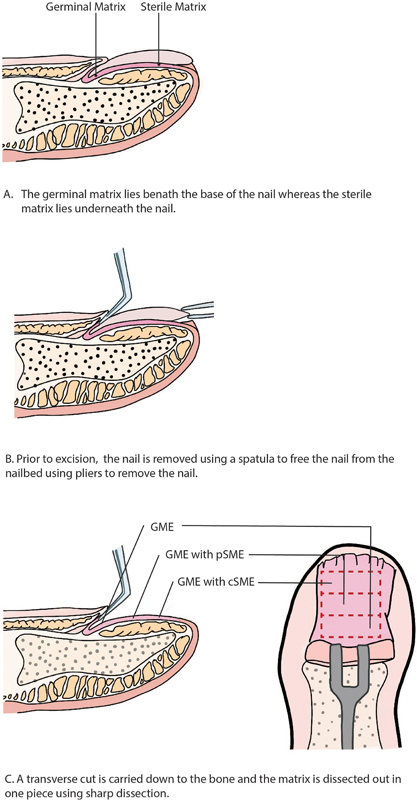
Illustration showing a longitudinal cross section of the nail tip and demonstrating the procedure.

With no established surgical technique for definitive treatment of the nails, three varying surgical procedures were performed on the patient's fingers and toes to identify which would be most effective in preventing painful regrowth. These techniques included GME alone, GME plus pSME, or GME plus cSME ( Table 1 , Fig. 2 ). All nail beds were closed with local skin grafts when unable to be closed primarily.

**Table 1:** A summary of involved digits, procedures, and outcomes

Digits	Procedure	Outcome
Right fingers 1, 2, 3, 4, and 5 Right finger 2	Germinal matrix excision Subsequent thinning ⅔ thickness of sterile matrix	Mild, slower hypertrophic nail growth in 1, 2, and 3. Hyperkeratinization approximating normal nail thickness in 4 and 5. Thinner but still hypertrophic nail
Right toe 1	Germinal matrix excision Subsequent sterile matrix excision; primary skin closure	Recurrent hypertrophic nail growth Primary skin healing, no nail growth
Right toes 2, 3, and 4	Germinal matrix excision	Hyperkeratinization approximating normal nail thickness
Right toe 5	Germinal and sterile matrix excision; primary skin closure	Primary skin healing, no nail growth
Left finger 1	Germinal matrix excision	Recurrent hypertrophic growth, slight improvement
Left finger 2, 3, 4, and 5	Germinal matrix excision	Mild, slower hypertrophic growth
Left toes 1, 2, and 3	Germinal matrix excision	Mild, slower hypertrophic growth
Left toes 4 and 5	Germinal and sterile matrix excision; primary skin closure	Primary skin healing, no nail growth

**Fig. 3:**
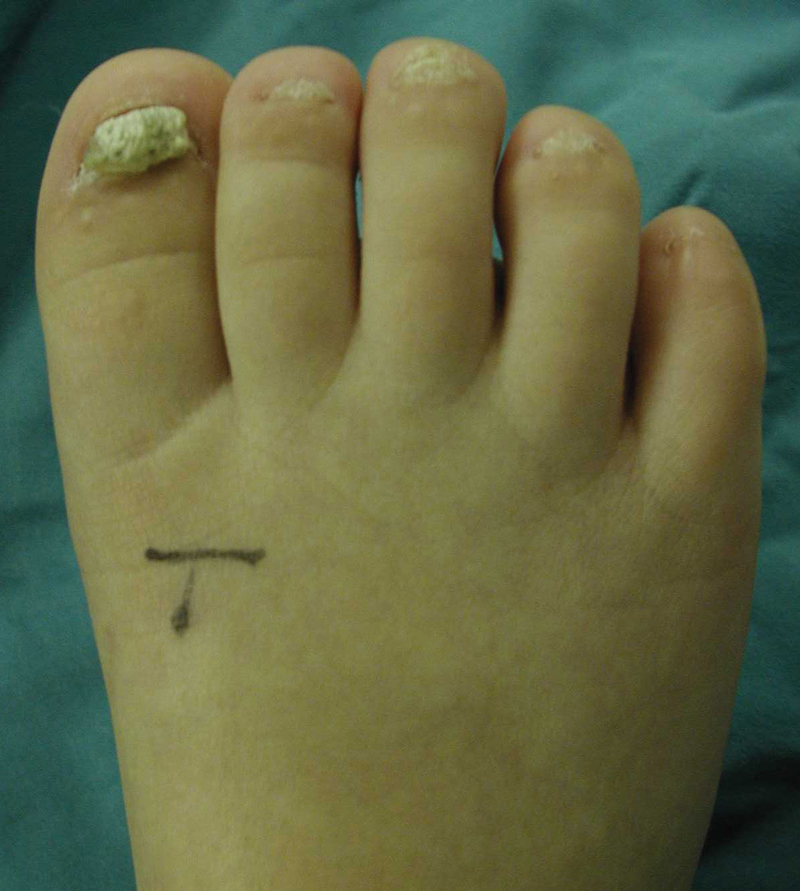
The right foot 8 months after germinal matrix resection on toes 1, 2, 3, and 4 and germinal matrix and sterile matrix resection on toe 5. Recurrence of the thickened nail on the great toe is noted. Subsequent resection of the sterile matrix of the great toe was curative. Toes 2, 3, and 4 show hyperkeratinization. Toe 5 has no nail regrowth.

The three toes treated with GME + cSME with proximal nail fold closure exhibited primary skin healing with no nail growth. This method of treatment was the most successful ( Table 1 ). The right hallux, treated with GME alone, exhibited slow but recurrent growth of the hypertrophic nail. Subsequent cSME and local skin closure resulted in no nail growth. The remaining toes, treated with GME alone, developed hyperkeratinization at a slower growth rate than before the operation ( Fig. 3 ).

**Fig. 4:**
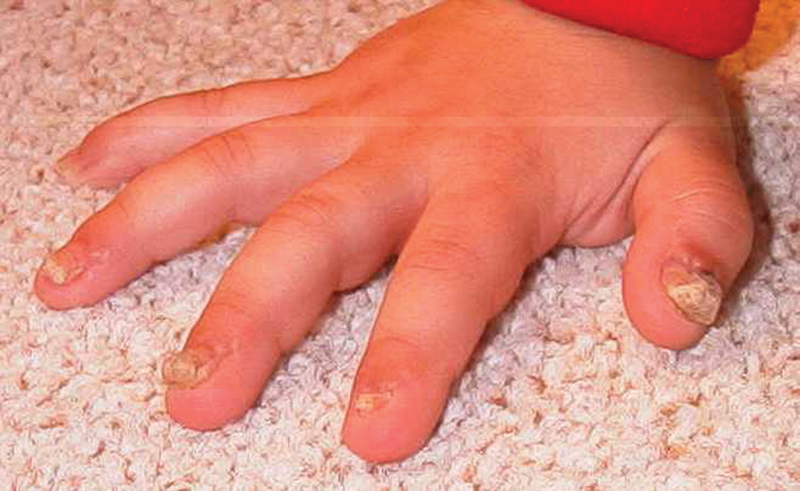
The right hand 8 months after germinal matrix resection of all five digits. The thumb, index, and middle show recurrence while the ring and little have hyperkeratinization approximating the thickness of a normal nail.

The thumbs exhibited bilateral thickened nail plate growth, though to a lesser degree than preoperatively. Additionally, the rate of growth was slower. The remaining two to five fingers exhibited nail plate hypertrophy but less than that of the thumbs ( Fig. 4 ). Nail growth was significantly slowed. Before surgery, nail trimming was necessary every month, but following GME, nail trimming was necessary every 3 to 6 months.

Due to recurrent hypertrophy, the thickness of the right index sterile matrix was thinned by two-thirds, resulting in a significantly thinner nail plate. Improvement was noted with this method, but the result was not ideal compared with GME + cSME. These results have persisted for 19 months following surgery.

## Discussion

Medical therapy to reduce hyperkeratosis is limited; however, several surgical techniques have been described to improve the function and appearance of digits with hypertrophic nail plates. The various methods address the germinal matrix and nail bed separately. Grinding and milling the nail plates to reduce nail plate thickness (under general anesthesia) followed by regular maintenance grinding of the nail plates (without anesthesia) effectively controls nail plate thickness. 5 The ongoing maintenance required for this control must be compared with the benefits of more permanent interventions. Daroach et al found that nail plate avulsion in combination with oral sirolimus significantly improved pain, but did not prevent the regrowth of abnormal nail plates and, consequently, was only of temporary benefit. 4 6 7 8 Control of the hypertrophic nail plates by amputation of the distal phalanx, while effective, is unacceptably extreme. 9

Cosman et al reported nail plate, GME, and pSME followed by full-thickness skin graft coverage with the remaining nail bed developing hyperkeratinization. 10 A more aggressive approach was reported by White and Noone in which the nail plate was excised with GME and then covered with a split-thickness skin graft. 11 No nail plate growth recurred. Thomsen et al treated different fingers with different surgical procedures. 6 They concluded that GME or destruction was sufficiently effective in treating the nail plate abnormalities and that cSME was unnecessary. The remaining nail bed developed hyperkeratinization, which they felt was acceptable. Vigorous curettage and fulguration of the germinal and sterile matrices produced smooth nail beds without nail plate growth on four of the six digits treated. Minimal hyperkeratosis developed on one nail bed, and a small spicule of recurrent nail plate grew on another. Preservation of the germinal matrix deep to the eponychium with excision of the distal germinal matrix and nail bed resulted in thickened nail plate regrowth. Excision was chosen in place of chemical germinal matrix ablation, seeing that ablation has reduced success in cases of recurrent nail growth. 12 13 A 2020 retrospective study by DeKlotz et al looked at 18 patients who had their nails removed and found that of the 18 individuals, 13 would recommend nail removal to others with PC. Most of the patients who had no regrowth of at least one nail would recommend that treatment. 14 This suggests that if nail removal is to be attempted, finding the process that would allow minimal regrowth would be of much benefit.

In this patient, the results implicate the involvement of both germinal and sterile matrix contributing to nail hypertrophy. In the toes treated with GME + cSME, no nail growth occurred. As Thomsen et al's study also suggests, the thickness of the sterile matrix remaining may correlate with the amount of hyperkeratinization. The presented results support this theory. 6 Initial postoperative hyperkeratinization of the right index finger was improved with thinning two-thirds of sterile matrix thickness. GME with cSME and closure of the defect secondarily or with a skin graft is a safe and effective option for treating nail hypertrophy in PC. This option showed to be the most definitive and successful treatment. An alternative that avoids skin grafting is full-thickness GME and pSME with thinning two-thirds thickness of the sterile matrix. Patients and their families should be counseled that some hyperkeratinization may result in preserving one-third thickness of the sterile matrix. The hyperkeratinization is significantly decreased compared with preserving the entire thickness of the sterile matrix. This option also does produce the appearance of a nail plate.

PC is a rare disease, and large cohorts are challenging to find. Although successful, the surgical management of this rare disorder is described in a single case, potentially limiting its applicability to all patients afflicted with PC. Therefore, inferences cannot be made on the most appropriate treatment for all PC patients. This patient was lost to follow-up prior to completing genetic testing. Therefore, the appropriateness of this procedure for specific genotypes of PC patients cannot be commented on. Further studies are needed to assess which exact mutations are appropriate for GME + cSME.

All procedures followed were in accordance with the ethical standards of the responsible committee on human experimentation (institutional and national) and with the Helsinki Declaration of 1975, as revised in 2008 (5). Informed consent was obtained from all patients for being included in the study. Additional informed consent was obtained from all patients for which identifying information is included in this article.
